# Unlucky punches: the *vulnerability-stress model* for the development of impulse control disorders in Parkinson’s disease

**DOI:** 10.1038/s41531-021-00253-z

**Published:** 2021-12-08

**Authors:** Hendrik Theis, Catharina Probst, Pierre-Olivier Fernagut, Thilo van Eimeren

**Affiliations:** 1 University of Cologne, Faculty of Medicine and University Hospital Cologne, Department of Nuclear Medicine and Department of Neurology, Cologne, Germany; 2 Université de Poitiers, INSERM, Laboratoire de Neurosciences Expérimentales et Cliniques, Poitiers, France

**Keywords:** Psychiatric disorders, Parkinson's disease, Human behaviour

## Abstract

Impulse-control disorders are commonly observed during dopamine-replacement therapy in Parkinson’s disease, but the majority of patients seems “immune” to this side effect. Epidemiological evidence suggests that a major risk factor may be a specific difference in the layout of the dopaminergic-reinforcement system, of which the ventral striatum is a central player. A series of imaging studies of the dopaminergic system point toward a presynaptic reduction of dopamine-reuptake transporter density and dopamine synthesis capacity. Here, we review current evidence for a *vulnerability-stress model* in which a relative reduction of dopaminergic projections to the ventral striatum and concomitant sensitization of postsynaptic neurons represent a predisposing (*hypo*dopaminergic) *vulnerability*. *Stress* (*hyper*dopaminergic) is delivered when dopamine replacement therapy leads to a relative overdosing of the already-sensitized ventral striatum. These alterations are consistent with consecutive changes in reinforcement mechanisms, which stimulate learning from reward and impede learning from punishment, thereby fostering the development of impulse-control disorders. This *vulnerability-stress model* might also provide important insights into the development of addictions in the non-Parkinsonian population.

## Introduction

Addiction is a global social, economic, and health problem. So far, there is no effective treatment and pathophysiology is insufficiently understood^1^. A key feature of addiction is the reduced ability to control behavior (e.g., drug intake, gambling), despite of their obviously harmful effects^2^. There are substance-related and non-substance-related addictions, such as pathological gambling, which are also called behavioral addictions or impulse-control disorders (ICDs). It is conceivable that for the development of ICDs, both predisposing traits and triggering mechanisms play a role. These traits and triggers may have identifiable biological substrates. The main pathophysiological mechanism in these disorders has been linked to disrupted dopamine homeostasis^2^. While longitudinal data covering the entire development of ICDs are almost impossible to come by in the general population, a unique opportunity is provided in Parkinson’s disease (PD). In a large multicenter study, Weintraub et al. demonstrated that ICDs occur as a side effect of dopamine replacement therapy (DRT) in 14% of PD patients^3–5^. Newer research points out that the incidence of ICDs in PD could be much higher (up to 46%)^6,7^. Therefore, the development of ICDs in PD could serve as a model for all addictions^8^ because seemingly mentally healthy PD patients develop ICDs in a very short period of time.

The most popular pathophysiological concept for the development of ICDs in PD is the so-called *overdose theory*: among the basal ganglia loops, the motor loop is mainly affected by neurodegeneration in PD. Hence, when DRT is administered, the dopaminergic tone in the motor loop is balanced, but the relatively intact limbic loop is overdosed, which leads to a *hyper*dopaminergic state in the ventral striatum^9,10^. The dopamine signal of the limbic basal ganglia loop modulates conditional learning and has motivational impact on a person’s behavior: via dopaminergic signaling, we learn implicitly to approach stimuli with positive outcomes (*Go*-Learning) and to avoid the opposite (*NoGo*-Learning)^11^. Accordingly, patients with ICDs show altered dopamine-modulated behavior in the form of impulsivity, risk proneness, and overengagement in rewarding behavior as well as deficits in inhibitory control^12–14^. While this concept is attractive, it does not explain why only a fraction of patients develop the ICD phenotype. Furthermore, findings of several imaging and rodent studies leave doubts about the *hyper*dopaminergic concept for ICDs in PD and hint at *hypo*dopaminergic changes in these patients, which may represent a premorbid biological vulnerability.

The aim of this review is to consolidate *hypo*dopaminergic findings with the *hyper*dopaminergic overdose theory in the form of a *vulnerability-stress model* for the development of ICDs in PD. In general, this model states that persons have an intrinsic vulnerability (e.g., genetic), leading in combination with an extrinsic stressor (e.g., life crisis, drug abuse) to the development of mental illness^15,16^.

Additionally, we want to shed light on the relationship between apathy and ICDs since both conditions might underlie comparable changes within the dopaminergic reinforcement system.

## Evidence for a premorbid *vulnerability* to ICDs

**Fig. 1:**
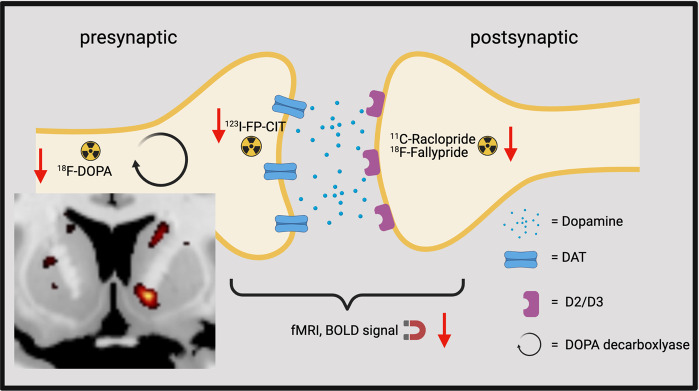
Imaging findings in ICDs in PD in the ventral striatum at the synaptic level. The red arrows symbolize reduced tracer uptake and reduced BOLD signal. The coronar brain slice shows the reduced dopamine synthesis capacity in the right ventral striatum^21^. (Created with BioRender.com).

Human imaging studies found several *hypo*dopaminergic changes in the ventral striatum of PD patients with ICDs: a reduced dopamine transporter (DAT) density in ^123^I-FP-CIT-SPECT^17–20^, a reduced dopamine synthesis capacity in ^18^F-DOPA-PET at rest^21^, a reduced BOLD activation at rest^22^, and a reduced D2/D3 receptor availability at rest^23–25^ (Fig. 1). These *hypo*dopaminergic changes could be inherited or acquired (e.g., by neurodegeneration). When considering general PD populations, Fazio et al. found that 36% of early PD patients had a reduced DAT density in the ventral striatum^26^ and early PD patients showed reduced dopamine synthesis capacity as compared with healthy controls in the ventral striatum^27^. Further evidence for the possibility of a premorbid vulnerability, that may be unrelated to dopaminergic neurodegeneration, comes from various studies reporting ICD development in non-PD populations (e.g., patients with fibromyalgia or prolactinoma) with DRT^28,29^. These *hypo*dopaminergic changes were also found in non-PD populations with behavioral or substance addictions: pathological gamblers^30^, alcoholics^31^, tobacco, and cannabis addicts^32^ showed a reduced DAT signal in the ventral striatum. Young people with internet addiction had a lower DAT binding^33^ and a reduced D2 receptor availability^34^ in the ventral striatum. Interestingly, a reduced DAT density was also found in healthy individuals with higher trait impulsivity^35^. Furthermore, a reduced dopamine synthesis capacity was also found in cocaine addicts^36^, cannabis users^37^, and binge eaters^38^.

An important study in drug-naive PD patients has shed further light on the question of whether these changes pre-existed before medication is started. In total, 31 de novo, drug-naive PD patients underwent DAT single-photon emission computed tomography (SPECT) and were screened for ICDs. After an average follow-up of 32 months, 11 had developed ICD symptoms without having any at baseline. These patients showed significantly lower baseline DAT binding ratios in the right ventral striatum, right anterior dorsal striatum, and right posterior putamen. Additionally, the severity of ICD symptoms at follow-up correlated negatively with baseline DAT availability^20^.

A reduced signal in DAT-SPECT in the ventral striatum could be due to reduced DAT density per dopaminergic terminal or due to a reduction of dopaminergic projections from the midbrain or a combination thereof. For further clarification, a recently published paper by our group used ^18^F-DOPA-PET to detect changes in dopamine synthesis capacity^21^. We found a negative correlation between the dopamine synthesis capacity and ICD severity in the ventral striatum at rest. Consequently, a predominant reduction of dopaminergic projections per se seems a more likely scenario. Interestingly, a postmortem study found no differences in tyrosine hydroxylase staining and α-synuclein load in the ventral striatum between PD patients with and without ICDs, indicating that the results from imaging studies could rather present functional changes than pure cell loss due to neurodegeneration^39^.

All in all, the results from the above-mentioned studies in PD and non-PD populations point to a weaker dopaminergic input to the ventral striatum as a premorbid *vulnerability* to develop ICDs.

### Beyond the *dopaminergic* reinforcement system

Although in this review we focus on dopamine, other neurotransmitter systems may play an important role for the development of ICDs. Serotonergic neurons project from the raphe nucleus to the ventral striatum and to the prefrontal cortex. Low serotonergic levels are associated with depression^40^ and trait impulsivity^41,42^, which in turn are associated with ICDs. Indeed, a PET study in de novo PD patients with apathy and depression did demonstrate a relative serotonergic denervation^40^. While these patients may be seen as “at-risk” to develop ICDs when medicated, there currently is no molecular serotoninergic imaging study in ICDs in PD. However, in non-PD binge eaters Majuri et al. found a reduced serotonin -transporter density in the ventral striatum^43^. Serotonin depletion in humans^44^ and rodents^45^ can lead to impulsive behavior, and polymorphisms in the serotonin-transporter protein are associated with addiction^46^. Moreover, perfusate serotonin increases dopamine release in the nucleus accumbens^47^. In sum, evidence is still lacking in PD, but a reduced serotoninergic input to the ventral striatum would be a plausible hypothesis. In a PET-study, a reduced μ-opioid receptor density^38^ was found in binge eaters in the ventral striatum. Furthermore, μ-opioid receptor stimulation in the nucleus accumbens amplifies hedonic wanting^48^. Interestingly, polymorphisms in the κ-opioid receptor were negatively associated with ICDs^49^. An animal study with microdialysis revealed that stimulation of these opioid receptors has an effect on striatal dopamine release^50^. Furthermore, an increase in glutaminergic projections from the prefrontal cortex to the ventral striatum leads to drug seeking^51^. Engeli et al. found a reduction of glutamate in the nucleus accumbens in cocaine addicts at rest and an increase in glutamate levels during cue-induced craving compared with healthy controls in a magnetic resonance spectroscopy paradigm^52^. All in all, changes in other neurotransmitter systems seem to influence the dopamine metabolism in the ventral striatum and might be associated with a *hypo*dopaminergic state in the ventral striatum as described above.

### Beyond the *ventral* striatum

Most of the imaging studies concerning ICDs in PD reported alterations in the ventral striatum. This mesolimbic reward circuit, including the ventral tegmental area and the nucleus accumbens, is crucial for mediating reward and the calculation of a reward prediction error^11,53^ and seems to be the key player for the development of addictions. An important feature of addiction and compulsion is that an action becomes habitual. The key player for habit formation is commonly seen in the dorsolateral, not the ventral striatum. Belin and Everitt could show that the ventral striatum is important for the initiation of drug seeking, while the dorsal striatum is more involved in sustaining it^53–55^. Interestingly, two studies found that a reduction in DAT density in the putamen and the anterior dorsal striatum^20^ as well as a reduced D2/D3 density in this area are associated with ICDs in PD^24^. In a recently published paper by our group, we found a negative correlation between dopamine synthesis capacity in the caudate and the severity of ICDs^21^. Other studies could show a reduced connectivity between the anterior cingulate cortex and the left putamen^56,57^. All in all, ICDs seem to be associated with alterations in dorsal striatum. A region that is more affected from neurodegeneration in PD than the ventral striatum. To sum up, we reason that the dopaminergic loss in the ventral striatum may be critical for the initiation of ICDs and that the dopaminergic loss in the dorsal striatum might play an important role for the long-term persistence of this behavior.

## Downstream consequences of reduced DAT and dopamine synthesis capacity

**Fig. 2:**
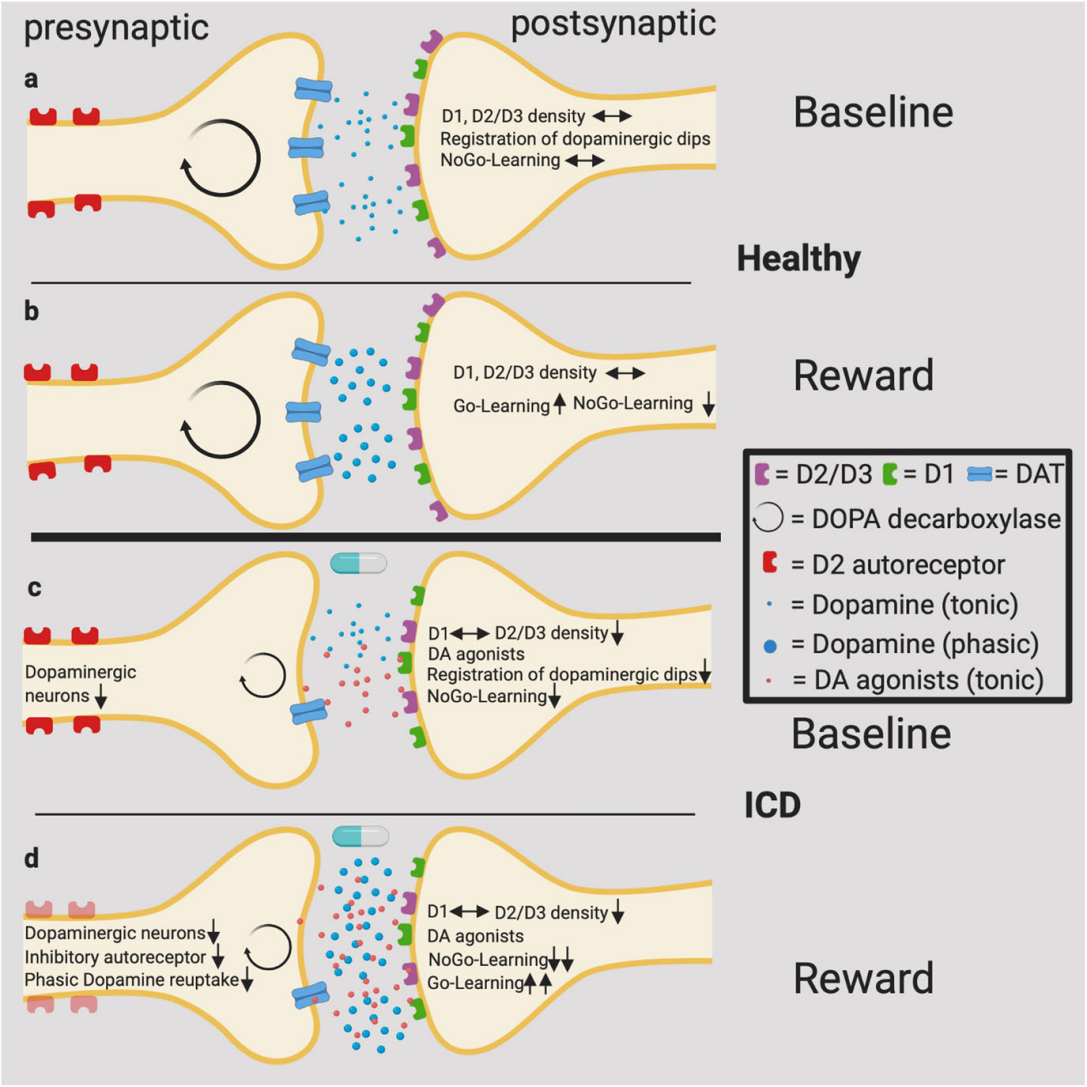
A *vulnerability-stress model* for the development of ICDs. **a** Normal tonic dopamine release and balanced density of D1 and D2/D3 receptors. Dopaminergic dips via D2/D3 can be registered, *NoGo*-Learning is possible. **b** A reward leads to a phasic dopaminergic burst in the striatum, which is followed by D1 stimulation and *Go*-Learning. The phasic dopamine release is stopped by inhibitory autoreceptors and dopamine reuptake. **c** The tonic dopamine release and the postsynaptic D2/D3 density are reduced. In combination with dopamine agonists, dopaminergic dips cannot be registered. *NoGo*-Learning is attenuated. **d** The ending of phasic dopamine release is disturbed because of a reduction of DAT density and a reduced activation of inhibitory autoreceptors. *Go*-Learning is emphasized, whereas *NoGo*-Learning is attenuated. (Created with BioRender.com).

Dopamine release in the striatum can conceptually be divided into two relatively independent forms, tonic and phasic dopamine release, which relate to tonic and phasic activities of dopaminergic neurons, and have distinguishable roles in learning from outcomes. On the one hand, an unexpected reward leads to a phasic dopamine release from the ventral tegmental area to the ventral striatum which is then followed by D1 receptor activation (*Go*-Learning). On the other hand, punishment or the omission of an expected reward leads to a dopaminergic dip and *NoGo*-Learning via D2/D3 receptors is fostered^11^. D1 receptors are activated after phasic dopamine release, whereas the activation of D2/D3 receptors (having a higher affinity to dopamine than D1 receptors) is dominated by tonic dopaminergic levels^58–60^. D3 receptors have the highest affinity to dopamine and are mainly located in limbic areas such as the ventral striatum^61^. Therefore, there have been speculations that D3 receptors are primarily involved in the development of ICDs. Interestingly, the influence of phasic dopamine release seems strongly affected by DAT activity, whereas the tonic dopamine release is mainly affected by the overall activity of a dopaminergic neuron population per se^62,63^. See Fig. 2A and B.

A possible downstream effect of reduced dopamine synthesis capacity could be a reduced tonic stimulation (i.e., occupation) of postsynaptic D2/D3 receptors. However, imaging studies found a reduced postsynaptic D2/D3 receptor availability in the ventral striatum at rest in PD patients with ICDs^23,24^. Then again, a reduced receptor availability measured by PET can have three different explanations: a reduction of receptor density, higher dopamine levels in the synaptic cleft (competing with the PET ligand), or a combination of both. In light of the reduced dopamine synthesis capacity at rest^21^, we may interpret a reduced D2/D3 receptor availability as primarily reflecting a reduction in receptor density. This fits well with a post mortem study, showing lower levels of D3 receptors in the ventral striatum of PD patients with ICDs. This study could not find changes regarding D2 receptors^39^. A reduced density of D2/D3 receptors in combination with an additional administration of dopamine agonists would hamper learning from negative feedback and lead as a consequence to ICDs (Fig. 2C)^11^. A concurrently reduced activation of presynaptic D2 autoreceptors, on the other hand, would lead to an increase in phasic dopamine, associated with a heightened propensity to reward-driven behavior (see also below).

Genetic studies point out that a reduction of DAT expression (polymorphism in DAT1 gene) is associated with addiction, PD, and ADHD^5,64,65^. Guo et al. found that individuals with the 10-repeat allele of the DAT1 had significantly more sexual partners^66^; and individuals with the 10-repeat allele had lower binding in DAT-SPECT as compared with patients with the 9-repeat allele^67^. Moreover, Volkow et al. found a reincrease in DAT density in abstinent cocaine addicts^68^. In animal studies DAT blocker enhances reactions to reward predicting cues^69^ and DAT knock-out mice show a greater locomotor sensitization to drugs, i.e., a greater progressive and persistent enhancement of the motor-stimulant effects of cocaine and ethanol^70^. Having in mind that the phasic dopamine release is mainly affected by the reuptake capacity of DAT in combination with the reduction of DAT density, as described above, we would measure more dopamine in the synaptic cleft as a consequence of a phasic dopamine release under reward conditions. In line with this hypothesis, imaging studies found a reduced D2 availability under reward conditions in PD patients with ICDs as compared with normal PD patients^23,71^. This would involve an increased dopamine release as well as a reduction in D2 receptors.

Furthermore, a chronic underexpression of DAT leads to a reduced function of midbrain D2 autoreceptors which may evoke higher extracellular dopamine levels^72,73^. According to a recently published review^74^, presynaptic D2 autoreceptors have three different possibilities to modulate dopamine metabolism: (1) reduction of the exocytotic dopamine release after a prior release, (2) regulating the dopamine uptake via an increase of DAT expression, and (3) downregulation of tyrosine hydroxylase (reduced filling of dopamine vesicles). Ray et al. found a reduced activation of these autoreceptors in PD patients with pathological gambling^75^, which could also explain increased phasic dopamine release. See Fig. 2D. Buckholtz et al. could show that healthy individuals with lower levels of D2 autoreceptor had a higher amphetamine-induced dopamine release^76^.

## The role of the prefrontal cortex—a loss of inhibitory top-down control

Besides from changes in striatal regions, the prefrontal cortex plays an important role for development of ICDs. The anterior cingulate cortex is crucial for error monitoring^77^ and behavioral adaptions after negative feedback^78^. The lateral orbitofrontal cortex is responsible for punishment-based decision-making^79^ and is important for suppression of previously rewarded behavior^80^. Voon et al. found a reduced BOLD activation of the anterior cingulate cortex in ICD in PD during risk-taking^14^. Another study reported a reduced activation of the anterior cingulate cortex and lateral orbitofrontal cortex in PD gamblers as compared with PD controls in H_2_O-PET^81^. Several other studies found a reduced connectivity between the anterior cingulate cortex and the ventral striatum^21,78^. Another PET study found a higher availability of D2 and D3 receptors in this area, which could indicate low levels of synaptic dopamine in PD patients with^75^. All in all, imaging studies point out that there is a diminished top-down control of inhibitory cortical areas in ICDs^5^.

## Dopamine replacement therapy—when it comes to *stress*

In case of L-DOPA, 7.2% of PD patients develop ICDs, 14% in case of dopamine agonists, and 17.7% in case of both^4^.

According to our proposed theory, due to *hypo*dopaminergic changes and the associated mechanisms, the system becomes vulnerable to relatively small alterations in dopaminergic levels. The system is adjusted to low levels of tonic dopamine and reduced D2 receptors. Then, as a consequence of DRT administration, the system becomes easily overdosed. Thereby, phasic effects are boosted and dips in dopamine release are drowned by the tonic D2/D3 overstimulation^82^.

Interestingly, patients taking dopamine agonists have twice the risk for ICD than patients taking L-DOPA alone. A reason could be the altered function of D2 autoreceptors in the midbrain^75^, which downregulate phasic striatal dopamine release. Chronic treatment with dopamine agonists may lead to a desensitization of D2 autoreceptors in the midbrain with consecutive dysregulation of phasic dopamine release^83^. Furthermore, dopamine agonists reduce the activity of inhibitory control areas in PD patients with ICDs, whereas they increase the activity in these areas in PD controls^81^. Likewise, dopamine agonszts diminish reward processing in the lateral orbitofrontal cortex during negative errors of reward prediction^82^.

In the same vein, a combination therapy with agonszts and L-DOPA will lead to the highest prevalence of ICDs because increased D1 effects (higher dopamine release because of L-DOPA and low D2 autoreceptor function) and D2/D3 overstimulation (dopamine agonists) are combined.

Astonishingly, time onset of ICD diagnosis after the initiation of DRT is highly variable (from 3 months up to 10 years)^84^. A reason could be the association between the cumulative dopamine agonist dose and the development of ICDs^6^. Likewise, Perez et al. could find a correlation between agonist dose and ICDs^85^ as opposed to Weintraub et al. using a different pharmacological model^4^. So, in every prone PD patient, there might be an individual (cumulative) dose threshold. After discontinuation of dopamine agonists, ICDs resolve in about 50% of the patients^6^. Two longitudinal studies could show an improvement of ICDs after reduction of agonists or a switch to L-DOPA^86,87^, whereas personality traits associated with ICDs persisted. So far, variable rates of relapse or remission are not fully understood and further research is needed.

## Is apathy the counterpart of ICDs?

Arguably, one could arrange the motivational spectrum of behavior in such a way that ICDs would be at the positive end and apathy at the negative end of the spectrum. Apathy generally is even more prevalent in PD patients than ICDs, including early stages of the disease^88^. While there potentially are multiple mechanisms leading to apathy, it is interesting that some forms of apathy are clearly temporally correlated with a reduction of dopaminergic stimulation. Apathy occurs following deep-brain stimulation, especially when DRT is reduced to a large degree^89^. Apathy can also be found as a part of dopamine-agonist withdrawal syndrome (DAWS). Intriguingly, dopamine agonist withdrawal in PD patients with DAWS almost always was preceded by ICDs, and in patients without ICDs, dopamine agonist withdrawal did not lead to apathy^90^. Additionally, when comparing PD patients with these two PD subgroups, overlaps in behavior were found^91,92^. Scott et al. showed in a recently published cohort study that more than a third of PD patients with apathy also suffer from ICDs. Interestingly, these were the patients with the longest disease duration^93^.

**Fig. 3:**
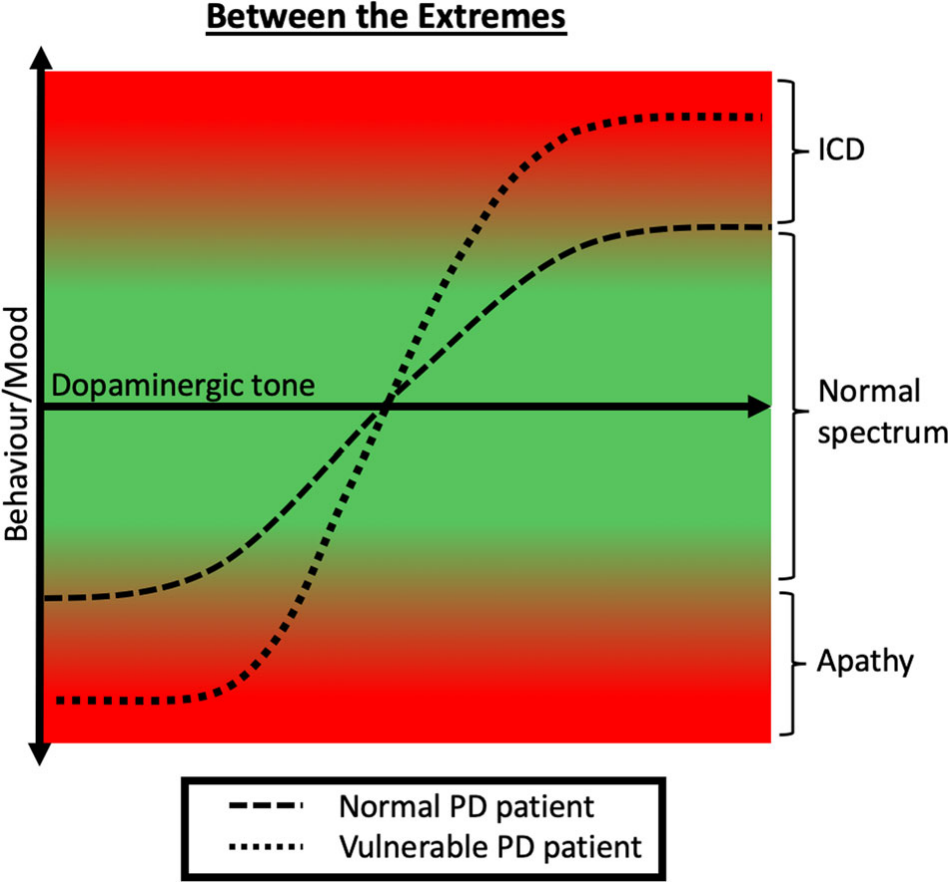
Between the extremes. Relation between dopaminergic tone and behavior illustrated as sigmoid curves in PD patients, with and without *vulnerability*. In vulnerable PD patients, the relationship between behavior and dopaminergic tone resembles a “flip-flop” switch scenario.

In addition, rodent studies^94,95^ as well as human imaging studies^96^ hint at a *hypo*dopaminergic state in the striatum predisposing for apathy. Therefore, apathy and ICD might share the same pathophysiological principle, i.e., *hypo*dopaminergic changes in the striatum, which then leads to either ICDs or apathy, depending on DRT. Sierra et al. use the term “Ying and Yang” of dopamine-dependent behavior^97^. Figure 3 describes hypothetical differences in dose–response relationships, implicating that vulnerable PD patients switch between the extremes in response to only small changes in dopaminergic medication. Additionally, it seems worth mentioning that not only might apathy share pathomechanisms with ICD but also dyskinesia (for detailed review see Voon et al.)^98^.

## Rodent studies—a possibility for validation of our *vulnerability-stress model*?

Rodent studies of the dopaminergic reward system show interesting insights into the development of ICDs. Lesions with 6-hydroxydopamine (6-OHDA) can imitate our theory of a premorbid vulnerability: Cardinal et al. produced a dopaminergic lesion in the nucleus accumbens with 6-OHDA, leading to impulsiveness in a delay-discounting task^99^. In other designs, dopaminergic lesions were set in the posterior VTA. After the submission of DRT, the animals showed impulsive behavior in a place-preference task^100,101^. Holtz et al. also used 6-OHDA to produce a dopaminergic lesion in the striatum. In a delay-discounting task, rats showed risk-taking behavior when pramipexole was administered. Interestingly, mirtazapine leads to a reduction of risk-taking^102^. In another design, 6-OHDA was administered at the substantia nigra. After surgery, animals took less from a rewarding sucrose solution. As a more general claim, rats became apathetic, which was fully reversed after the intake of pramipexole^94^, which supports our theory-comparable changes within the dopaminergic reinforcement system in apathy and ICD described above.

Interesting insights into the effect of dopaminergic medication, *stress* in our model, can be derived from chemogenetics. “Designer receptor exclusively activated by designer drugs” (DREADDs) can be used to activate or inactivate certain types of dopaminergic receptors. Boender et al. injected a D1-activating DREADD in the nucleus accumbens of rats, leading to an increased intake of sucrose pellets, which was annulated by the administration of a D1 antagonist^103^. Zhu and colleges injected D2 receptor activating and inhibiting DREADDs in the nucleus accumbens of rats. D2 activation reduced locomotion and running, whereas D2 inhibition had the opposite effect^104^.

In all, rodent studies—despite their limitations in comparability—corroborate and validate biological concepts of the *vulnerability-stress model* of ICD development.

## Conclusions

We discuss a hypothetical model of *hypo*dopaminergic changes in the ventral striatum that would act as a biological *vulnerability* toward addictive behavior. These alterations predispose the dopaminergic system (*vulnerability*), which, in combination with DRT (*stress*), leads to ICDs. As the most likely scenario, a reduction of dopaminergic projections in combination with a reduced DAT density and autoreceptor function results in adjustment processes at the postsynaptic membrane. Furthermore, it comes to a diminished top-down control of inhibitory cortical areas. As a consequence, DRT overwhelms the prone system. So, a combination of a premorbid *vulnerability* and *overdosing* could lead to ICDs in PD and can be seen as a *vulnerability-stress model*. It is tempting to speculate that similar biological processes may underlie other drug or non-substance addictions in the non-PD population.

Apathy is associated with a reduced DAT density in the dorsal striatum, whereas patients with ICDs have also a reduction of DAT in the ventral striatum. We assume that apathy and ICDs go along with a *hypo*dopaminergic state in striatal regions and therefore with an increased sensibility to DRT. There is an overlap in patients suffering from both ICDs and apathy.

## Limitations

The model of *hypo*dopaminergic changes in the ventral striatum, leading to a *vulnerability* for DRT and thereby to ICDs, is only hypothetic and, of course, a simplification of the complex development of ICDs. So, there are some limitations to consider. In this review, we mainly shed light on the so-called dopaminergic reinforcement system, but also other neurotransmitters, as mentioned above, play an important role.

Additionally, we do not have de novo data concerning the postsynaptic membrane. We do not know whether the reduced D2 availability might predate the presynaptic changes. Hence, theoretically, the model described above could be vice versa. Changes in D2 density could lead to a hypodopaminergic state in the striatum.

Furthermore, imaging data do not always point in the same direction: One study found an increase in dopamine synthesis capacity in impulsive PD patients^105^. In addition, Boileau et al. could not find differences in D2/D3 availability between pathological gamblers and healthy controls^106^. Similarly, the results of genetic studies in PD with ICDs are not consistent^107^. Altogether, despite many converging results around a premorbid biological vulnerability, more rigorous studies with larger samples are needed to consolidate the genetic and molecular features of this *vulnerability*.

## Future directions

It would be interesting to measure D2 receptor availability and DAT density in de novo PD patients and after the development of ICDs under DRT. With this approach, it would be possible to check if postsynaptic changes also predate the development of ICDs. In addition, it would be interesting to measure dopamine synthesis capacity under reward conditions since all existing ^18^F-DOPA-PET studies measure baseline dopamine synthesis capacity. This would help to classify the reduced D2 receptor availability under reward conditions. Furthermore, a validation of our *vulnerability-stress model* in a rodent model combined with PET imaging would be of great interest for the understanding of ICDs in PD in particular but also for the development of addiction in general.

### Reporting summary

Further information on research design is available in the Nature Research Reporting Summary linked to this article.

## Supplementary information

Reporting Summary

## Data Availability

No datasets were generated or analyzed during the current study.
